# Effectiveness of “Hand Hygiene Fun Month” for Kindergarten Children: A Pilot Quasi-Experimental Study

**DOI:** 10.3390/ijerph17197264

**Published:** 2020-10-04

**Authors:** Lorna Kwai Ping Suen, Janet Pui Lee Cheung

**Affiliations:** Squina International Centre for Infection Control, School of Nursing, The Hong Kong Polytechnic University, Hong Kong; shampoo_miao@yahoo.com.hk

**Keywords:** pre-school, kindergarten, hand hygiene, education, quasi-experimental

## Abstract

Early childhood is a formative period during which healthy habits are developed, including proper hand hygiene practices. The aim of this quasi-experimental study was to determine the effectiveness of a 4-week series of educational sessions that consider the cognitive developmental stage of children on increasing their knowledge and promoting hand hygiene practices. The intervention group (*n* = 33) observed the hand hygiene program, whereas another group served as the waitlist control (*n* = 20). Creative activities were planned for the illustration of hand hygiene concepts in terms of “right moments”, “right steps”, and “right duration”. Hand sanitizer coverage was evaluated using a hand scanner. After the intervention, the experimental group had higher knowledge level toward hand hygiene than the control group (*p* < 0.001). Significant improvements in hand hygiene performance at the left palm and dorsum (*p* < 0.05), right palm (*p* < 0.05), and overall hand coverage (*p* < 0.05) were observed in the experimental group. The study demonstrated that the knowledge and proper hand hygiene (HH) practice of children can be positively influenced by the use of an age-appropriate education program. The results of this study have implications for school health educators and parents for promoting HH practices among children at home and at the school level.

## 1. Background

Upper respiratory tract and gastrointestinal infections are common infectious diseases in childcare settings [1,2,3]. Children under center care are more susceptible to contracting pathogens than children who stay at home due to frequent physical contact [3,4].

The hands are the most common transmission medium of many infectious conditions particularly among children playing, eating, and sleeping closely together in a school setting [5]. Children are prone to place things in their mouths, eat with their hands, and pick their noses [6,7,8]. Proper hand hygiene (HH) practice can effectively reduce the incidence of upper respiratory infection; diarrhea; and/or hand, foot, and mouth disease (HFMD) among children in childcare settings, thereby reducing absenteeism associated with these illnesses [1,2,5,9]. HFMD, for example, is a widespread pediatric disease that has become an endemic childhood disease in East and Southeast Asia. HFMD can develop several complications, such as viral meningitis, encephalitis, poliomyelitis-like paralysis, or death [10,11]. The World Health Organization and the United Nations Children’s Fund have jointly launched the “Hand Hygiene for All Global Initiative” recently to emphasize the importance of HH for combating coronavirus disease 2019 (COVID-19) and a range of infectious conditions [12].

HH is a general term referring to any action of hand cleansing, using soap and water or any hand hygiene products, such as alcohol-based hand rub [13]. Good HH practices can remove or destroy pathogens and thus prevent their transmission and break the chain of infection. However, poor HH compliance is evident in young children, and most of them do not perceive HH as important to their health and wellness [4,14,15].

Early childhood is a formative period when healthy habits, including proper HH practices, are developed and instilled [16]. Therefore, teaching children to take responsibility for their own health is important. The major aim of health education for children should be to help them to make the right choices or decisions related to their health behavior. A quasi-experimental study conducted in Hong Kong found that kindergarten students with strong parenting and proper HH compliance can help to reduce flu-like absenteeism among students [17]. Another observational study assessed the changes in knowledge and handwashing practices after providing a thematic lecture on hygiene for kindergarten children and reported that the proportion of participants capable to complete handwashing procedures is low [18]. As such, interactive and interesting age-appropriate teaching activities are suggested to be adopted.

A conducive learning environment can provide safety, comfort, and interest in learning [19]. Therefore, a well-designed program on HH that considers the cognitive developmental stage of children is important. As evidence that considers the cognitive developmental stage of children and supports the effectiveness of an age-appropriate program on HH is lacking, a program named “Hand Hygiene Fun Month” was launched and evaluated. The aim of this quasi-experimental study is to determine the effectiveness of a 4-week series of educational sessions that consider the cognitive developmental stage of children on increasing their knowledge and promoting the HH practices. The findings of this study would provide school health educators with some recommendations for promoting HH education in child care settings.

## 2. Methods

### 2.1. Participants

This quasi-experimental study was conducted in an international kindergarten in Hong Kong. Participants were level 3 kindergarten children (equivalent to the preschool kindergarten year of the USA). More than 370 children aged 2–6 years attend this whole-day service kindergarten, which has a staff-to-student ratio of 1:10. The target learners were level 3 kindergarten children aged about 5–6 years. The reasons for targeting this age group are to establish proper HH behaviors since childhood and consider the higher cognitive level of these kindergarten students than that of lower level classes. Students that have lesions on hands, a history of allergy to hand sanitizer or eczema, and those that declined to participate were excluded. Sixty-three students from three classes were assessed for inclusion in the study. Fifty-three students were finally recruited after excluding those who declined to participate (*n* = 8) or those with eczema (*n* = 2), with a recruitment rate of 84%. Twenty-four students from two classes were assigned to the experimental group, and 15 students of the same class were assigned as waitlist controls through convenience sampling.

### 2.2. Instruments

The sociodemographic data that included age and gender were collected. The below measurements were taken from the participants in both groups at baseline (T0) and after the “Hand Hygiene Fun Month” (T1) was completed. The consistency of the measurements was determined by evaluating the inter-rater reliability between the two researchers involved in the assessment.

#### i) Hand Hygiene Knowledge Questionnaire for Children

A 12-item “Hand Hygiene Knowledge Questionnaire for Children” was designed by the research team for the evaluation of the knowledge level on HH of the participants. The questionnaire consisted of different situations illustrated by 12 cartoon pictures for assessing children’s knowledge of situations where HH should be performed. These situations include after sneezing, before or after toilet time, before or after meal, after play time, after singling, before sleep, after shower, after changing shoes, after taking off facial masks, and before watching television (Supplementary 1). The validity of the questionnaire was examined by an expert panel of seven members that consisted of two infection control nurses, one nurse specialist with a pediatric background, one school principal, one kindergarten teacher, and two school nurses. A content validity index of 93% was achieved. Test–retest reliability was performed on eight children at a two-week interval. The value for intraclass correlation coefficient (single measure) of the knowledge questionnaire was 0.625 (95% confidence interval = −0.070–0.912, *p* < 0.05), which indicated moderate reliability [20] of this instrument. The score ranged from 0 to 12, and a higher score indicated better knowledge of HH.

#### ii) Use of Hand Scanner for Assessing the Coverage of Hand Sanitizer

**Figure 1:**
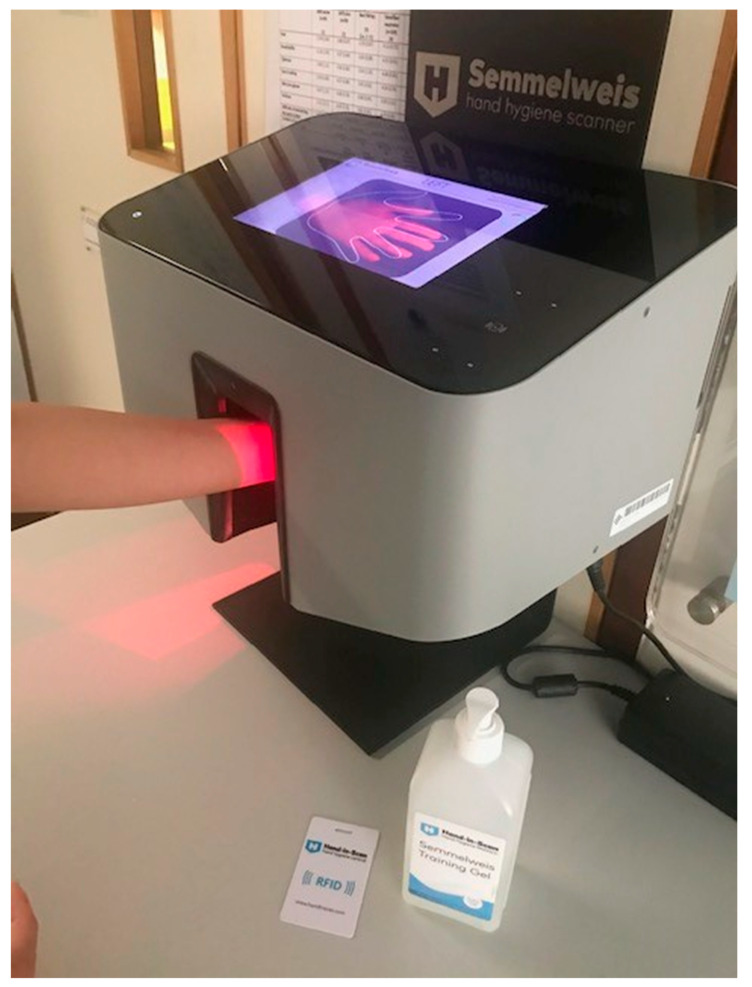
‘Hand-in-Scan’ device.

Hand sanitizer coverage on hands was evaluated using a “Hand-in-Scan” Semmelweis Hand Hygiene Scanner (HandInScan Kft, Debrecen, Hungary. Model: HINST20E3WS0P01) (Figure 1). The children were instructed to perform HH following their usual technique by using hydroalcoholic gel that contains a fluorescent agent (Semmelweis Training Gel, Hand Hygiene Training Solution, Budapest, Hungary). Children were asked to get an adequate amount of gel to cover the hands, usually by squeezing one pump (~2 mL) of the gel for hand disinfection. The participants then placed their hands into the “Hand-in-Scan” that could capture the image of the hands in seconds. The screen of the device displayed silhouettes of both hands to show the participants where they should position their hands. A radiofrequency identification card with a code for identification of the participant was provided during data storage.

Areas covered by hand sanitizer were displayed in green color, and the missed areas were shown in red. The hand regions under assessment included the palms, back of hands, finger webs, back of fingers, thumbs, and finger tips (wrists were not included as this region was not included in the template for analysis) (Supplementary 2).

### 2.3. Procedures

The study was conducted from January to May 2019. Before the study, we conducted a site visit to familiarize ourselves with the practices and settings of the kindergarten regarding HH. The play areas, study areas, and washroom facilities were examined. Washrooms were equipped with automatic water supply systems. Handwashing signage, liquid soap, and an enriched foam alcohol-free hand sanitizer (Rubbermaid-TC®, RM-750593, Rubbermaid Commercial Products, New York, NY, USA) were provided for children.

This work was a quasi-experimental study using a series of educational sessions as intervention. The intervention group received the program on HH during the “Hand Hygiene Fun Month”. Another group served as the waitlist control and received the program after post-measurement was completed in both groups.

The project has been reviewed and approved by the Ethics Committee of the Hong Kong Polytechnic University (HSEARS20181204003). Parental consent for participating in this workshop was sought. Participation to this survey was voluntary, and confidentiality of the data was strictly observed.

#### Elements of the Hand Hygiene Educational Program

A series of educational sessions of the “Hand Hygiene Fun Month” contained four weekly teaching sessions. Each teaching session was delivered in 20 min. The details of the activities in the workshop are described below.

#### i) First Session

The activity in the first session focused on the “right moments” of HH practice. Considering the unique setting of kindergartens, our research team proposed the “5 Moments” for HH practice for school children, emphasizing the need for HH practice (1) before tea time; (2) before entering the classroom; (3) after playtime; (4) after touching dirty surfaces or body fluids, such as changing shoes, sneezing, or coughing; and (5) after using the toilet. The concepts of performing HH at the “right moments” was instilled by using the “Hand Hygiene Knowledge Questionnaire for Children” that consisted of different situations illustrated by 12 cartoon pictures for teaching (the details of this questionnaire are above).

**Figure 2:**
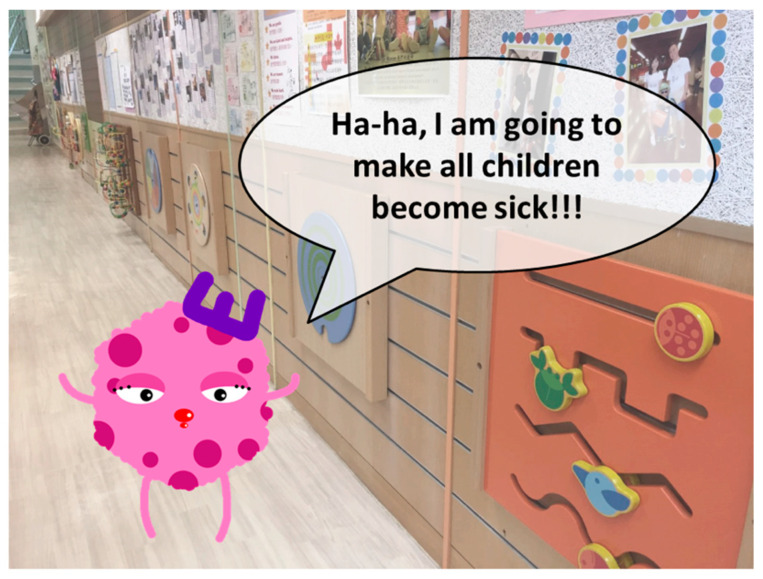
Screenshot of the animated video.

The students in the experimental group watched an animated video (Figure 2) illustrating the relationship between germs and diseases, and the participating kindergarten was used as the background. This video could help children to understand that germs exist everywhere in their living environment. HH can be used as a “weapon” to destroy the germs, and children are the “little soldiers” that should use this “weapon” at the “right moments”. Teaching children through the use of an animated video can arouse their interest in the learning process.

#### ii) Second Session

**Figure 3:**
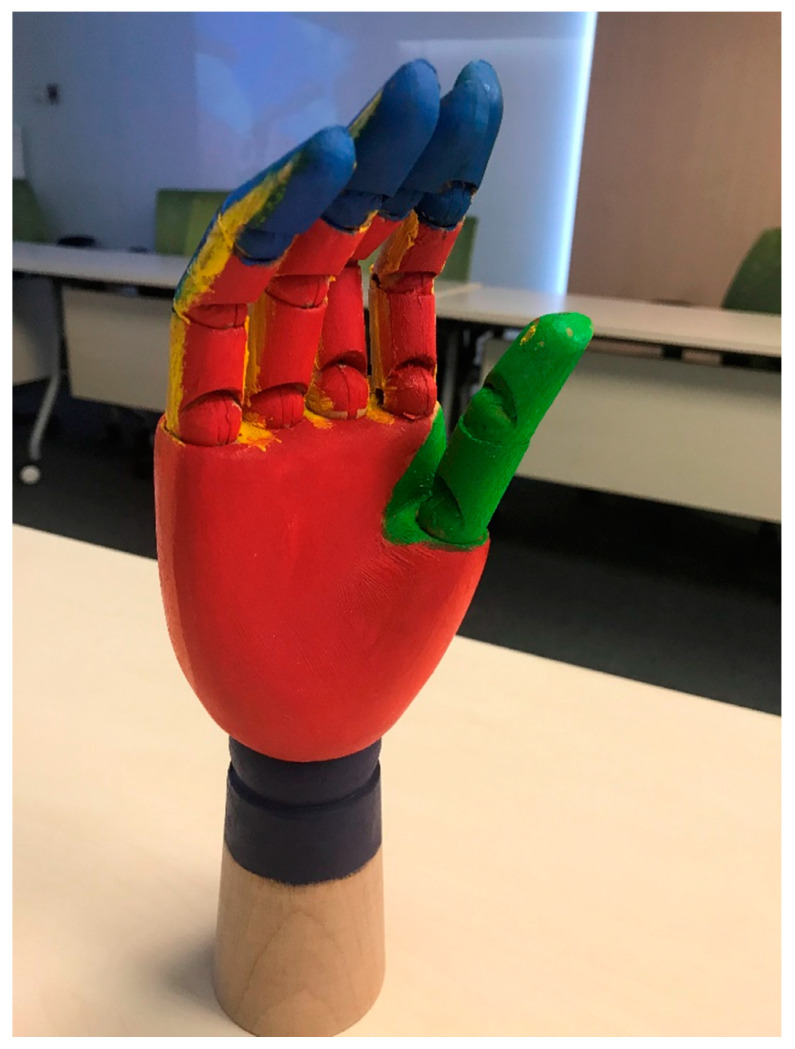
Wooden hand model painted with 7 rainbow colors.

**Figure 4:**
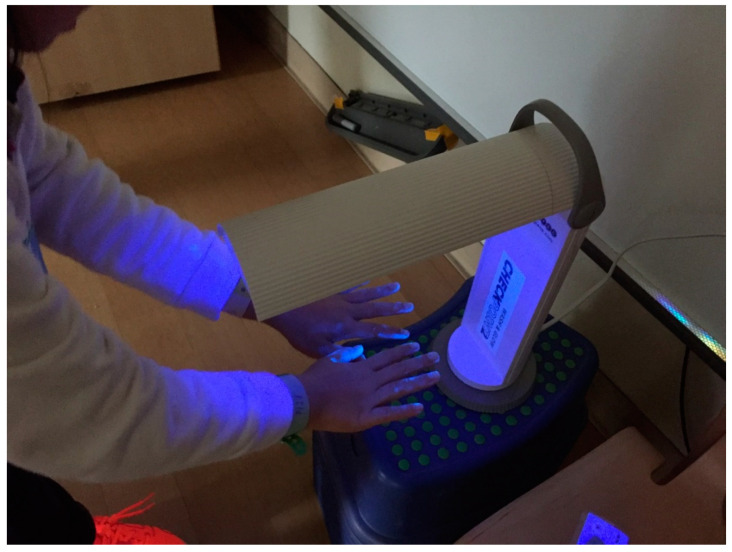
Hands were examined using an ultraviolet lamp (CheckPoint).

The focus of this session is the importance of the “right steps” to perform HH. A wooden hand model painted with seven rainbow colors corresponding to the seven regions of the hand was used (Figure 3). The regions of the hands included the palms, back of hands, finger webs, back of fingers, thumbs, finger tips, and wrists. To enhance the children’s learning effect, we instructed them to play a musical game by passing around a small toy sprinkled with Glo Germ™ powder that contained florescent properties. When the music stopped, the child holding the toy had to name one of the mentioned hand regions. Stickers were given as incentives to those who named the regions correctly. After a few rounds of the games, the “germs” on their hands were examined using an ultraviolet lamp (CheckPoint, 220–240 V/50 Hz; Glow Tec Ltd., London, UK) (Figure 4). This game can enable the children to understand how germs are spread through daily personal contacts and activities and emphasize the importance of HH practice. Moreover, hand drying using paper towels or hand dryers and the proper use of hand sanitizers were highlighted.

#### iii) Third Session

**Figure 5:**
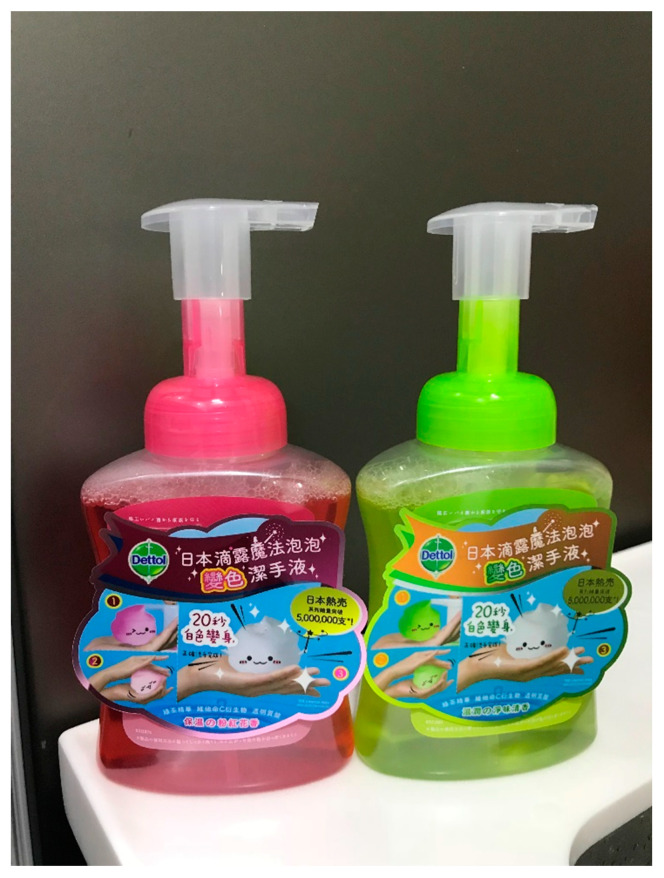
Color foaming hand wash.

This teaching session focused on the “right duration” when performing HH. At the beginning of the session, each child was asked to do a handprint on an agar plate and then taught about the importance of the duration of performing HH. The children were asked to perform subsequent hand washing using a color foaming hand wash (Dettol®, Reckitt Benckiser, Hong Kong) (Figure 5). According to the manufacturer’s instruction, the color of the foaming hand wash changes from its original color (either pink or green) to white after rubbing on the hands for 20 s. Therefore, it could serve as a reference for children so that they can spend enough lathering time before rinsing. After hand washing was performed, the second handprint on the agar plate was taken again. The purpose of performing the agar plate experiment was to instill the concept that germs on the hands can be removed through proper HH. The results of the agar plate were shown to the students after culture for one week at room temperature.

#### iv) Fourth Session

**Figure 6:**
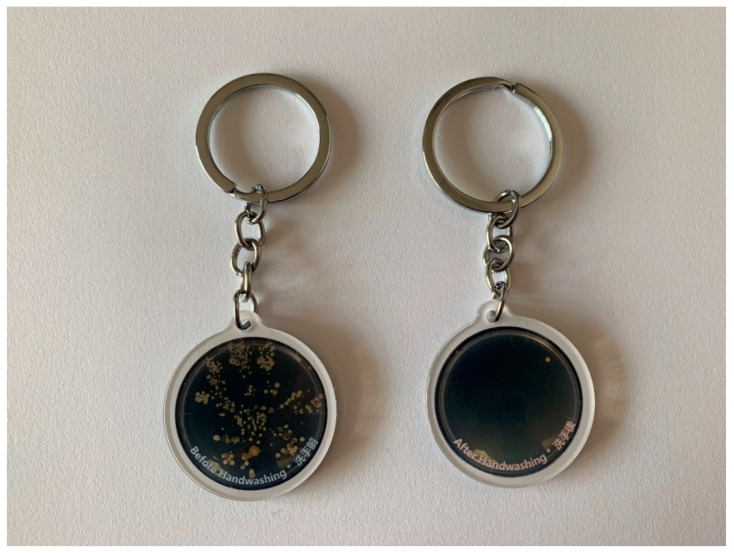
Key chain with handprint photos.

In this final session, the students were given the opportunity to view the results of their respective agar plate cultures. By comparing the handprints taken before and after performing HH, the children could understand the effect of hand washing on reducing bacterial count on the hands. A plastic key ring with photographs of the agar plate culture results (Figure 6) and a bottle of color foaming hand wash were distributed to children as souvenirs so that they would be encouraged to spread the HH messages to their families and continue to practice proper HH at home. The children were encouraged to make a commitment to be “little soldiers” to defeat germs using HH as “weapons” in this closing session.

### 2.4. Data Analyses

Descriptive statistics for demographic characteristics, knowledge, and practice associated with the HH of children using hand sanitizers were presented. The Wilcoxon signed-rank test was used to determine within-group comparison. The Mann–Whitney *U*-test was used in the evaluation of the between-group differences of hand coverage (%) with regard to the use of hand sanitizer. For categorical variables, such as regions of hands covered by hand sanitizer (missed versus covered regions) and the knowledge toward HH at different situations (correct versus incorrect answer), either the x^2^ test or Fisher’s exact test were used where appropriate. Gender differences in HH performance and group difference in the overall knowledge score were determined via the Mann–Whitney *U*-test. SPSS version 25.0 (IBM Corporation, Armonk, NY, USA) was used for all statistical analyses. All statistical tests were two-sided, with a significance level set to 0.05.

## 3. Results

**Figure 7:**
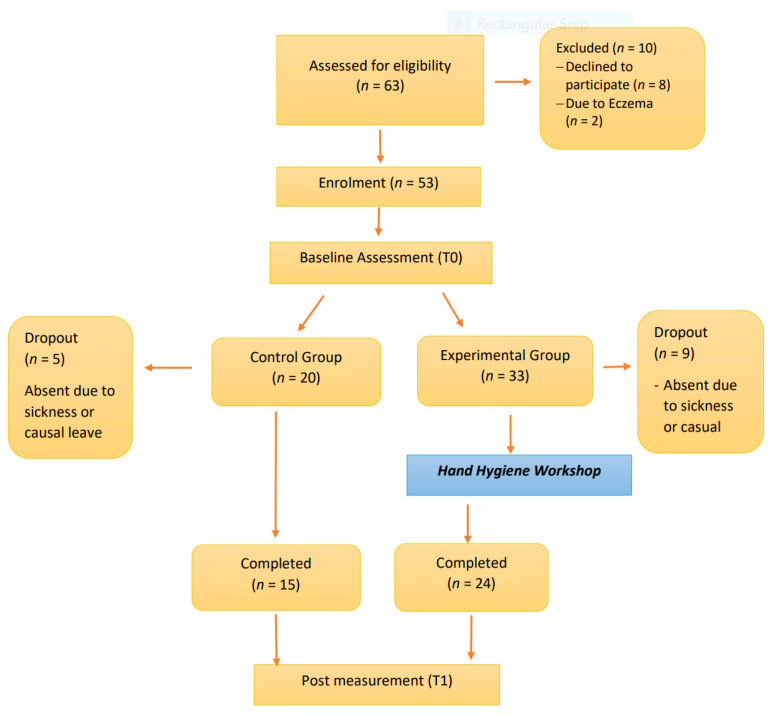
Flowchart of study.

Among the 53 recruited participants, 14 dropped out from the study (dropout rate: 26.4%) because of either sickness or causal leave. A total of 39 students were entered into the analysis. These students comprised 59.0% (*n* = 23) females and 41.0% (*n* = 16) males, with a mean age of 5.81 ± 0.33. The flow diagram of the participants is illustrated in Figure 7.

### 3.1. Knowledge Level Towards Hand Hygiene Behaviors

Some participants were not aware that HH should be performed or not in certain occasions, such as before toilet, after meal, and after changing shoes. The experimental group had significantly higher scores than the control group after the intervention (11.71 versus 9.87, *p* < 0.001; Table 1).

### 3.2. Hand Coverage by Hand Sanitizer

Significant improvements in HH performance at the left palm and dorsum (*p* < 0.05), right palm (*p* < 0.05), and overall hand coverage (*p* < 0.05) were observed in the experimental group after the intervention (Table 2). With regard to the different regions of the hands, the most commonly missed regions when using hand sanitizers were the back of hands (94.9%), thumbs (92.3%), back of fingers (69.2%), finger webs (61.5%), and finger tips (56.4%). The children in the experimental group generally had a higher coverage of hand sanitizer in all hand regions than those in the control group after the intervention, and a statistically significant difference was noted in the thumb region (*p* = 0.031) (Table 3). Female students generally performed better than male students, although the group differences were not significant (Table 4).

## 4. Discussion

According to the different stages of cognitive development proposed by Piaget (1896–1980), a famous Swiss philosopher and psychologist, children aged between 4 and 7 year are at the intuitive thought of the preoperational stage [21]. Children at this age range tend to be curious around the environment. They begin using primitive reasoning, have interest in reasoning, and are eager to know why things are the way they are [22]. A number of creative activities accorded with the developmental stage of students were planned for the illustration of important HH concepts in terms of “right moments”, “right steps”, and “right duration”.

The children who participated in the activities of the “Hand Hygiene Fun Month” were found to have an increase in their knowledge level after the program. The use of cartoon pictures helped to instill the concepts of performing HH at the “right moments”, especially at occasions that were previously ignored, such as after changing shoes. Some children also expressed that HH must be performed before toilet time and after meal. Even though performing HH at these moments is not mandatory, we have emphasized to the students the principles of performing HH when needed, e.g., before toilet time and after meal.

### 4.1. Age-Appropriate Creative Approaches for Teachings Important HH Concepts

Story-telling using an animated video was used in the first session for icebreaking and promoting a warm atmosphere conducive to the students’ learning. Children aged 4–6 years love listening to stories about animals, especially when the animals were represented by child-friendly images [23].

The use of a wooden hand model painted with seven rainbow colors corresponding to the seven regions of the hand emphasized the importance of the “right steps” when performing HH. Much attention has been paid to cleaning the hands regions that were previously overlooked, and a significant improvement was observed in the thumb region. According to Piaget’s Stages of Cognitive Development, the complex abstract thoughts of children at the preoperational stage (aged 2–7) are still difficult [21]. Apart from using the colored hand model to ease memorization, a simplified approach for learning the “right steps” for HH may be considered. Lee et al. [24] devised a simplified five-step hand washing technique for children with mild intellectual disability. This technique focuses on washing (1) between fingers, (2) back of hands, (3) back of fingers, (4) finger tips, and (5) thumbs.

The technique was found to be effective in enhancing HH practice and reducing the spread of infectious diseases among the participants.

Glo Germ™ powder and fluorescent gel are widely used in teaching concepts about germs [25,26] and known to be safe. To enhance children’s learning effect, we instructed them to play a musical game by passing around a small toy sprinkled with Glo Germ™ powder that contained florescent properties. The powder was used to simulate “germs” on the children’s hands. By passing around this small toy among their peers, students could learn how “germs” can be easily transmitted from person to person during group games.

To reinforce the importance of “right duration” when performing HH, we instructed the children to perform hand washing using a color foaming hand wash (Dettol®). Although the color of the foaming hand wash changes from its original color (either pink or green) to white after rubbing on the hands in less than the recommended duration (i.e., 20 s), such kinds of hand soap can serve as a reference for children for them to spend a long time lathering before rinsing. Moreover, the researcher discussed the germ concept with the students after the agar plate experiment. The distribution of a plastic key ring with photos of agar plate culture results and a bottle of color foaming hand wash to children as souvenirs can encourage them to spread HH messages to their family members and continue to practice proper HH at home.

Gender differences on hand coverage using hand sanitizer were observed among participants. Female students generally performed better than male students. Gender disparity on hand hygiene knowledge and practices has been frequently reported, and females generally have better overall knowledge of HH even at adulthood [27,28,29]. Moreover, a significant number of males ignored handwashing when they were in a hurry, nobody was in the washroom, or they only urinated [30].

Using direct observation for monitoring HH compliance is time-consuming, and being under the notion of being observed may alter HH behavior [4,31]. The “Hand-in-Scan” Semmelweis Hand Hygiene Scanner (HandInScan Kft, Debrecen, Hungary. Model: HINST20E3WS0P01) as an objective device was used in the evaluation of hand sanitizer coverage on the participants’ hands. A previous quasi-experimental study demonstrated that the visual feedback of the hand scanner had a positive effect on the HH performance of a group of nursing students [28].

### 4.2. Limitations

The small sample size in this study may limit the generalizability of the findings. Larger samples at multicenters should be considered in future replication of studies. Given that proper HH practices have the potential to decrease the incidence of infectious diseases, such as upper respiratory, gastrointestinal tract infection, and HFMD, future studies should consider using absentee records as one of the indicators for evaluating program effectiveness. In addition, the involvement of teachers and parents in future studies can help to reinforce proper behaviors on HH at school and home environments.

Although a remarkable improvement in the HH practice of the children in the intervention group was observed, the study failed to monitor whether the behaviors were sustained over time. Therefore, frequent reminders to children and long-term monitoring of program effectiveness should be conducted.

## 5. Conclusions

The study demonstrated that knowledge and proper HH practice of children can be positively influenced by the use of an age-appropriate education program. Children can learn effectively through creative activities that consider the developmental stage of children. The results of this study have implications for school health educators and parents for promoting HH practices among children at home and at the school level.

### Implications for Public Health

The results of this study demonstrated that the activities of the “Hand Hygiene Fun Month” can increase the knowledge level and enhance proper practices on HH among students in the child care setting. Age-appropriate activities were effective in teaching and enhancing the learning ability. The findings of this study suggest some implications for school health educators. The study demonstrated that the knowledge and proper HH practice of children can be positively influenced by the use of an age-appropriate education program. Creative activities were planned for the illustration of hand hygiene concepts in terms of “right moments”, “right steps”, and “right duration”. Children can learn effectively through creative activities that consider the developmental stage of children. This proposed program only consisted of four teaching sessions and can be easily integrated in the existing packed curriculum at preschool education. This program allows children to associate feelings of fun and establish the habit of HH. The earlier good healthy habits are developed in children, the more likely it is that these habits can be continued as children mature [32]. In addition, proper HH education should be an integral strategy to prepare for schools reopening to reduce the risk of coronavirus transmission during the COVID-19 epidemic.

## Figures and Tables

**Table 1:** Knowledge level between groups towards hand hygiene.

Variables	Experimental Group	Control Group	*p*-Value ◈
	(*n* = 24)	(*n* = 15)	
In which of the following situations, hand hygiene must be performed?	Correct responses		
	%		
After sneezing ※			
T0	100	100	---
T1	100	100	---
After singing			
T0	83.3	46.7	0.031
T1	95.8	86.7	0.547
Before toilet time			
T0	52.4	53.3	1
T1	95.8	60	0.008 **
After toilet time※			
T0	100	100	---
T1	100	100	---
Before meal ※			
T0	95.2	100	1
T1	100	93.3	0.385
After meal			
T0	33.3	26.7	0.734
T1	87.5	33.3	0.001 **
After play time ※			
T0	75	93.3	0.216
T1	95.8	93.3	1
Before sleep			
T0	58.3	80	0.295
T1	95.8	86.7	0.547
After shower			
T0	79.2	93.3	0.376
T1	100	86.7	0.142
After changing shoes ※			
T0	66.7	46.7	0.318
T1	100	53.3	<0.001 ***
After taking off facial masks ※			
T0	100	86.7	0.142
T1	100	100	---
Before watching television			
T0	83.3	93.3	0.631
T1	100	93.3	0.385
Total correct (score) ❖			
T0	9.29 (1.73)	9.20 (1.66)	0.7
T1	11.71 (0.69)	9.87 (1.51)	<0.001 ***

T0: Baseline; T1: Post measurement. ◈ Fisher’s exact test (when expected cell sizes less than five)/chi-square test (as appropriate); ❖ Mann–Whitney *U* test for continuous data; ※ hand washing should be performed; --- no statistics are computed because the variable is a constant; ** statistically significant at *p* < 0.01; *** statistically significant at *p* < 0.001.

**Table 2:** Within-group and between-group comparisons of hand coverage (%) using hand sanitizer.

Variables	Experimental Group (*n* = 24)		Control Group (*n* = 15)		Comparisons between Groups
	Mean (SD)	Within-group *p*-Value #	Mean (SD)	Within-group *p*-Value	*p*-Value ❖
Palm (Left) (%) T0 T1	92.67 (6.35) 94.88 (4.67)	0.361	92.45 (6.29) 89.29 (7.51)	1.000	0.851 0.017 *
Dorsum (Left) (%) T0 T1	66.79 (26.37) 85.09 (17.56)	0.001 **	46.64 (30.65) 64.65 (30.36)	1.000	0.057 0.030 *
Palm (Right) (%) T0 T1	92.29 (5.78) 97.36 (2.44)	0.001 **	93.92 (3.20) 92.01 (8.99)	0.865	0.583 0.018 *
Dorsum (Right) (%) T0 T1	70.58 (27.79) 75.42 (22.60)	0.304	44.32 (30.62) 54.54 (34.34)	0.427	0.015 * 0.126
Overall hand coverage (%) T0 T1	80.58 (14.34) 88.19 (10.14)	0.006 **	69.34 (15.83) 75.12 (17.12)	0.394	0.053 0.010 *

T0: Baseline; T1: Post measurement; # Wilcoxon signed ranks test; ❖ Mann–Whitney *U* test for continuous data; * statistically significant at *p* < 0.05; ** statistically significant at *p* < 0.01.

**Table 3:** Regions of hands covered by hand sanitizer.

Variables	All (*n* = 39)	Experimental Group (*n* = 24)	Baseline			Post-Measurement		
			Control Group (*n* = 15)		All (*n* = 39)	Experimental Group (*n* = 24)	Control Group (*n* = 15)	
	%	%	%	*p*-Value ◈	%	%	%	*p*-Value ◈
Palms Missed Covered	2.6 97.4	0.0 100.0	6.7 93.3	0.385	5.1 94.9	4.2 95.8	6.7 93.3	1.000
Back of hands Missed Covered	94.9 5.1	91.7 8.3	100.0 0.0	0.514	84.6 15.4	79.2 20.8	93.3 6.7	0.376
Finger webs Missed Covered	61.5 38.5	54.2 45.8	73.3 26.7	0.317	74.4 25.6	66.7 33.3	86.7 13.3	0.263
Back of fingers Missed Covered	69.2 30.8	58.3 41.7	86.7 13.3	0.083	76.9 23.1	75.0 25.0	80.0 20.0	1.000
Thumbs Missed Covered	92.3 7.7	87.5 12.5	100.0 0.0	0.271	82.1 17.9	70.8 29.2	100.0 0.0	0.031 *
Finger tips Missed Covered	56.4 43.6	58.3 41.7	53.3 46.7	1.000	51.3 48.1	45.8 54.2	60.0 40.0	0.514

◈ Fisher’s exact test (when expected cell sizes less than five)/chi-square test (as appropriate); statistically significant at *p* < 0.05.

**Table 4:** Hand coverage using alcohol-based hand rub by gender.

Variables	Male (*n* = 16)	Female (*n* = 23)	Between Group Comparison
	M ± SD	M ± SD	*p*-Value ❖
Before intervention			
Palm (Left) (%)	89.73 ± 6.18	94.18 ± 5.81	0.030 *
Dorsum (Left) (%)	51.37 ± 30.50	61.39 ± 29.47	0.125
Palm (Right) (%)	90.90 ± 5.34	93.63 ± 4.28	0.252
Dorsum (Right) (%)	54.76 ± 32.24	59.51 ± 30.35	0.857
Overall hand coverage (%)	71.69 ± 15.96	77.18 ± 15.31	0.160
After intervention			
Palm (Left) (%)	92.35 ± 7.56	93.37 ± 6.41	0.868
Dorsum (Left) (%)	72.70 ± 30.73	78.73 ± 21.93	0.856
Palm (Right) (%)	92.90 ± 9.04	96.31 ± 3.07	0.432
Dorsum (Right) (%)	57.50 ± 35.39	72.33 ± 24.62	0.283
Overall hand coverage (%)	78.87 ± 18.06	85.19 ± 12.00	0.505

Abbreviation: M, mean; SD, standard deviation. ❖ Mann–Whitney *U* test; * statistically significant at *p* < 0.05.

## Supplementary Materials

The following are available online at https://www.mdpi.com/1660-4601/17/19/7264/s1, Supplementary 1: Hand Hygiene Knowledge Questionnaire for Children; Supplementary 2: Sample of the ‘Hand-in-Scan’ report.
