# Supplementary material for: Effectiveness of “Hand Hygiene Fun Month” for Kindergarten Children: A Pilot Quasi-Experimental Study
Source: Int J Environ Res Public Health. 2020 Oct 4;17(19):7264. doi: 10.3390/ijerph17197264 (PMC7579510; doi:10.3390/ijerph17197264)
Supplement: Supplementary file 1 [file ijerph-17-07264-s001.zip › suppl_1_questionnaire.pdf]

Hand Hygiene Knowledge Questionnaire for Children

In which following situations, you MUST perform hand hygiene? Circle the correct answers.

Student Code:____________________

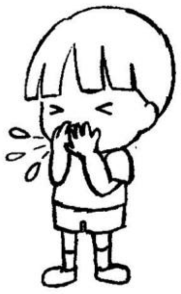

After Sneezing

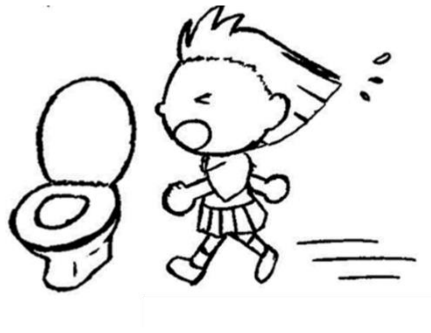

Before Toilet Time

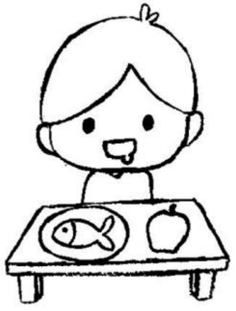

Before Meal

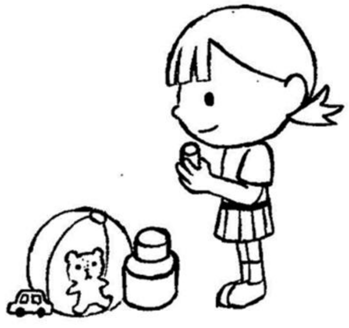

After Play Time

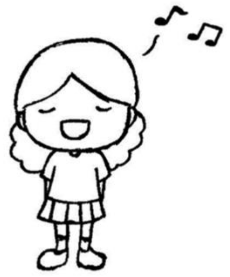

After Singing

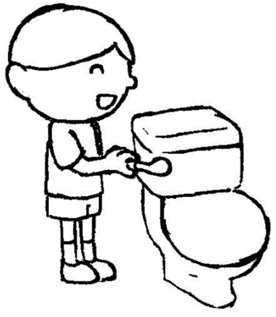

After Toilet Time

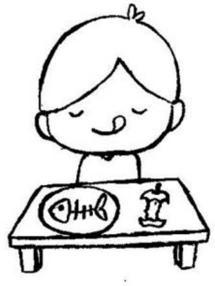

After Meal

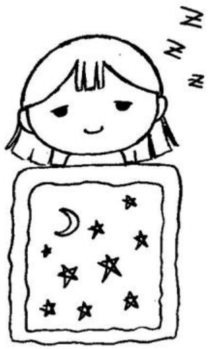

Before Sleep

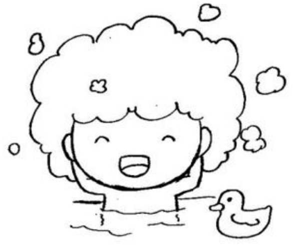

After Shower

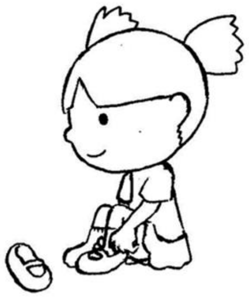

After Changing Shoes

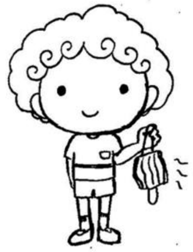

After Taking Off Facial Masks

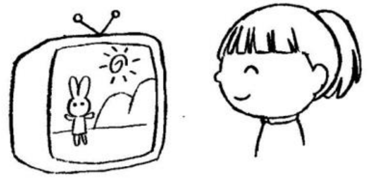

Before Watching TV
