# Supplementary material for: Effectiveness of “Hand Hygiene Fun Month” for Kindergarten Children: A Pilot Quasi-Experimental Study
Source: Int J Environ Res Public Health. 2020 Oct 4;17(19):7264. doi: 10.3390/ijerph17197264 (PMC7579510; doi:10.3390/ijerph17197264)
Supplement: Supplementary file 1 [file ijerph-17-07264-s001.zip › suppl_2_report.pdf]

Measurement Report

Semmelweis
Hand Hygiene Scanner

Name	4173 University of Polytechnic	Result (Target Coverage: 95%)
Organization	The Hong Kong Polytechnic University	65.4% FAILED
Department	School of Nursing	
Occupation	student	
Wrist	Not included in the result.	
Date	07/01/2019 02:17 PM	

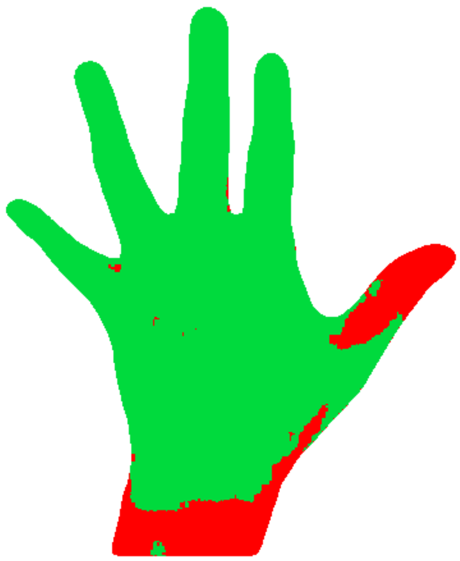

Left dorsum coverage: 92.2%

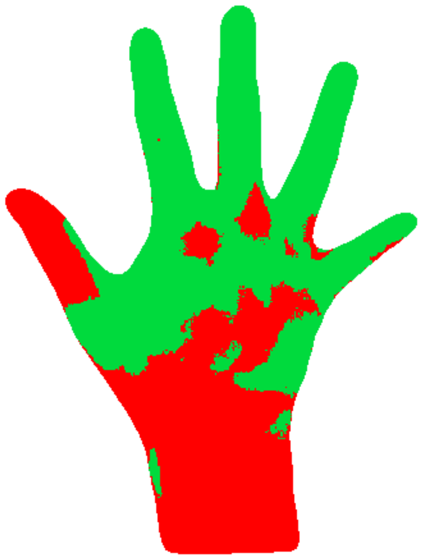

Right dorsum coverage: 65.4%

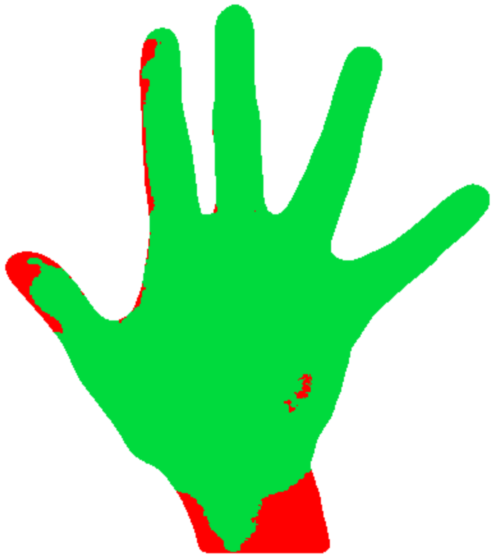

Left palm coverage: 96.7%

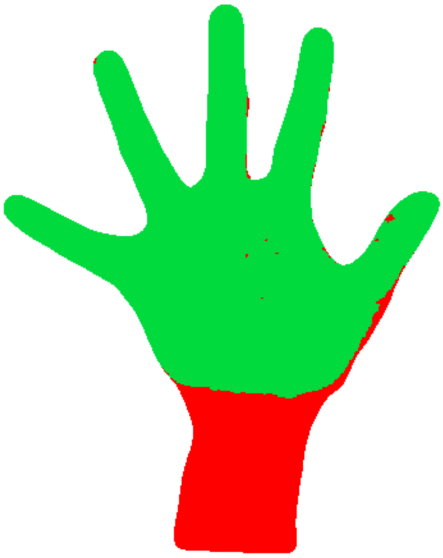

Right palm coverage: 88.7%

Regional
Evaluation

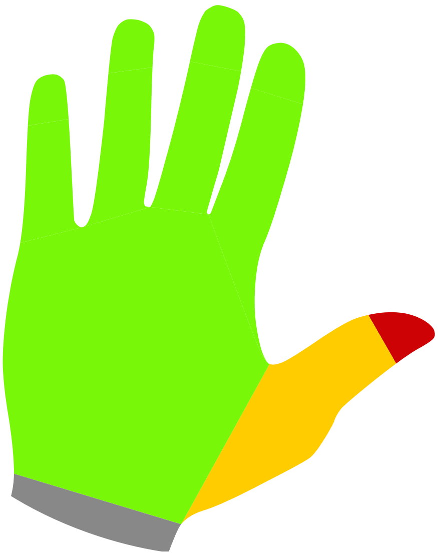

Left dorsum coverage

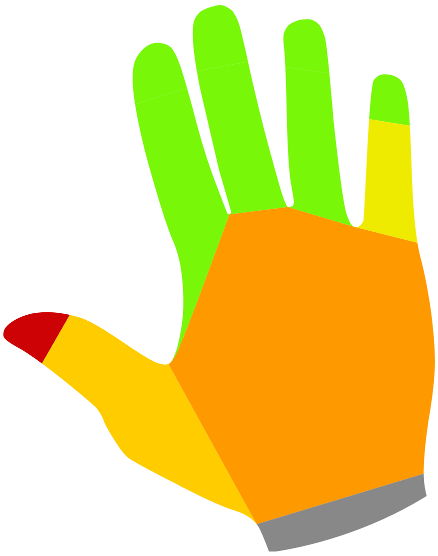

Right dorsum coverage

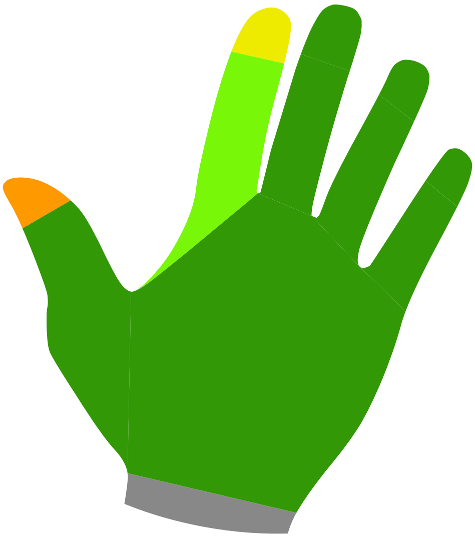

Left palm coverage

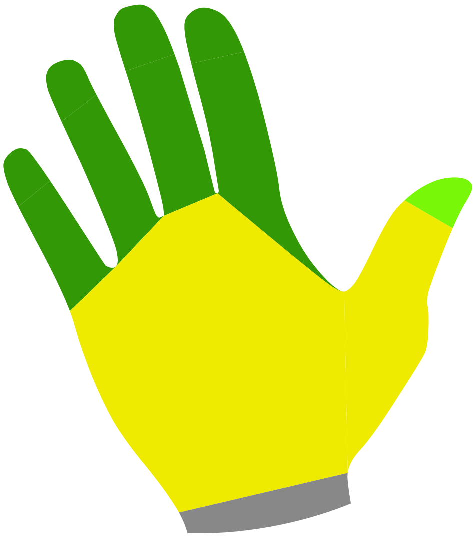

Right palm coverage

Most frequently missed

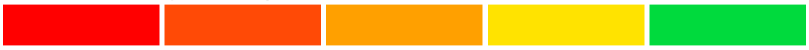

Least frequently missed
